# Air blast injuries killed the crew of the submarine *H*.*L*. *Hunley*

**DOI:** 10.1371/journal.pone.0182244

**Published:** 2017-08-23

**Authors:** Rachel M. Lance, Lucas Stalcup, Brad Wojtylak, Cameron R. Bass

**Affiliations:** 1 Naval Surface Warfare Center Panama City Division, Code E15 Underwater Systems Development and Acquisition, Panama City, Florida, United States of America; 2 Duke University Department of Biomedical Engineering, Durham, North Carolina, United States of America; 3 Duke University Medical School, Durham, North Carolina, United States of America; 4 Bureau of Alcohol, Tobacco, Firearms, and Explosives, Wilmington, North Carolina, United States of America; University of Florida, UNITED STATES

## Abstract

The submarine *H*.*L*. *Hunley* was the first submarine to sink an enemy ship during combat; however, the cause of its sinking has been a mystery for over 150 years. The *Hunley* set off a 61.2 kg (135 lb) black powder torpedo at a distance less than 5 m (16 ft) off its bow. Scaled experiments were performed that measured black powder and shock tube explosions underwater and propagation of blasts through a model ship hull. This propagation data was used in combination with archival experimental data to evaluate the risk to the crew from their own torpedo. The blast produced likely caused flexion of the ship hull to transmit the blast wave; the secondary wave transmitted inside the crew compartment was of sufficient magnitude that the calculated chances of survival were less than 16% for each crew member. The submarine drifted to its resting place after the crew died of air blast trauma within the hull.

## Introduction

### The submarine *HL Hunley*

**Fig 1:**
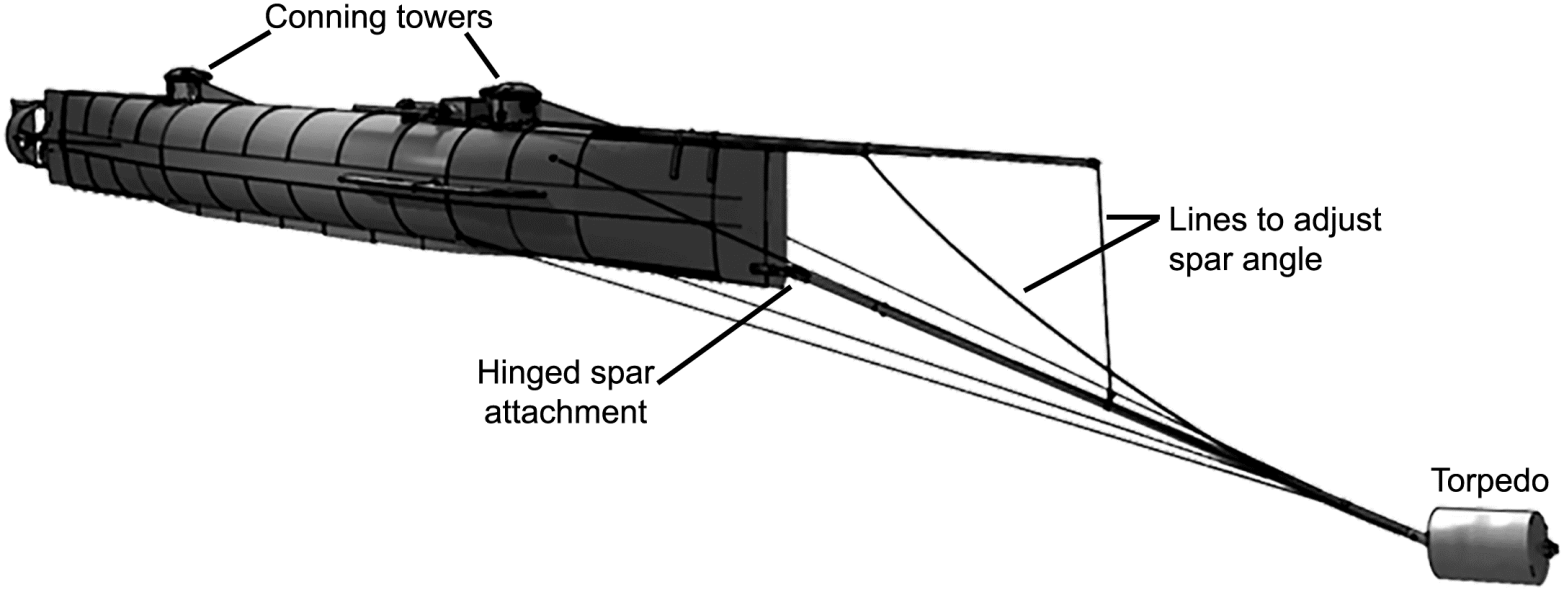
The *HL Hunley* as it would have appeared in attack position on the evening of February 17, 1864. Image courtesy Michael Crisafulli of The Vernian Era. More renderings and details of the construction of the *Hunley* can be found at http://www.vernianera.com/Hunley/.

The *Hunley* sank the Union ship *Housatonic* and killed 5 Union soldiers by setting off a black powder torpedo against the ship’s hull on the evening of February 17, 1864. The narrow, tapered submarine was 12 m (40 ft) long with a maximum width of only 1.2 m (4 ft) [1]. It was shaped out of the wrought iron boiler of a previous ship, and carried a crew of 8 men (Fig 1).

The vessel’s commander could see out the fore conning tower and was responsible for navigation, while the remaining crewmen powered the vessel’s propeller from the inside using a hand crank [2, 3]. At the other end of a hinged 16-foot spar was firmly bolted the *Hunley’s* torpedo, a copper torpedo of the common Singer’s design type filled with 61.2 kg (135 lbs) of black powder and fitted with a pressure-sensitive trigger (S1 Fig)[4].

The *Hunley* was raised from the ocean floor in 2000, and conservation efforts have been ongoing since [5, 6]. The skeletal remains of the crewmembers were found seated at their respective stations, with no physical injuries or apparent attempts to escape [7–9]. The conning towers, which formed the only path of escape, were closed with the aft tower still securely locked [10]. The bilge pumps were not set to pump out water [11]. The keel ballast weights, which could be released from within the boat, remained firmly attached [1, 12].

The two large holes discovered in the bow and side of the hull were determined not to be the cause of sinking by analysis of the sediment layers within, which showed that both breaches occurred long after the sinking, and no additional damage was found to the hull that provided an explanation. The holes were determined to have occurred at a later date because analysis of the types and quantity of sedimentary materials, including marine macrofauna, showed strata of sediment deposition that permitted analysis of the general patterns of sediment accumulation over time within the hull [13]. These strata indicated that the holes were not present during the vessel’s initial time underwater. The pattern of damage of the holes was determined to have been caused by a combination of galvanic corrosion, stresses from riveted seams, and erosion from ocean currents [14].

Both of two previous primary theories of sinking, suffocation and damage to the hull from arms fire, have been found to be implausible in recent publications [15, 16]. In addition, evaluation of the attack showed that the *Hunley* likely drifted before finally sinking [16].

### Underwater explosions

The increase in both the density and the speed of sound in water means that when explosions occur underwater, the resulting shock and pressure waves travel more efficiently and further than they do in air [17, 18]. The most critical behaviors of underwater shock waves follow traditional scaling laws since peak overpressure, duration, and impulse scale with the overall length scale of the experiment [17, 19]. However, it is more informative and convenient to describe the output of the charge via the equations provided by Hopkinson scaling, also referred to as the principle of similitude [20]. The Hopkinson scaling equations for peak pressure and time constant for underwater blast are shown as Eqs (1) & (2) [17, 21].

Pmax=kpressure(W13R)αpressure (1)

θ=ktimeW13(W13R)αtime (2)

k = scaling constants

α = decay constants

W = charge weight

R = distance from the center of the charge

P_max_ = peak positive overpressure

Θ = time constant of initial decay (μsec)

Blast interactions with structures can be prohibitively complex to test experimentally, so these tests are often performed both in air and in water by scaling down the size of the experiment according to the relevant dimensionless parameters [17, 22]. G.I. Taylor first described the pi groups that dictate the behavior of an underwater blast wave hitting a solid structure with air behind that structure, primarily in an attempt to predict damage to ships from TNT depth charges [23]. Taylor’s dimensional analysis and subsequent studies by other groups concluded that the amount of momentum transferred into a structure by a blast wave can be scaled by size if the time-relevant parameters of the blast wave are also scaled [22, 23]. Dimensionless parameters dictating the amount of momentum transferred are shown as Eqs (3) & (4).

Taylor, 1963 [23]

$$\varepsilon =\frac{\rho c}{mn\text{sin}\theta }$$ (3)

ρ = density of medium in front of the structure

c = speed of sound in medium in front of the structure

m = areal density of the structure

n = inverse time constant of decay of the blast wave

θ = angle between the direction of wave propagation and the structure surface

Kambouchev et al, 2006 [24]

$$\beta_{s}=\frac{\rho_{s}U_{s}t_{i}}{\rho_{P}h_{P}}$$ (4)

ρ_s_ = mass density of the material in front of the structure

U_s_ = propagation speed of the incident blast wave

t_i_ = duration of the incident blast wave

ρ_p_ = mass density of the structure

h_p_ = structural thickness

Momentum transfer into a structure produces motion of that structure, and rapid initiation of motion can create a shock wave off the structure’s back surface [22, 25, 26]. Transmitted blast waves have been observed computationally behind structures and experimentally measured behind armor [22, 27]. Computational simulations confirm that backface pressure response is the product of rapid motion of the structure wall in response to the original external blast, and because the motion response of the structure increases for stronger blasts, so will the magnitude of the transmitted blast [22, 25]. Such a backface wave means that, even if the original blast wave is largely reflected at the front material interface of the structure, it is possible that people behind the structure could still be injured or killed by transmitted shock from a sufficiently large charge at a sufficiently close range without overt damage to the structure.

### Black powder

Black powder is a low explosive, a volatile blend of crushed charcoal, sulfur, and either sodium or potassium nitrate [28]. Unlike high explosives, which have burn rates faster than the speed of sound, black powder deflagrates rather than detonates. Variables such as grain size, powder density, and even the type of wood used to make the charcoal can potentially have noticeable effects on powder performance because of their impact on burn rate [29–31]. Tests of modern black powder have shown comparable performance in both burn rate and pressures produced to cannon-grade Union powder from the Civil War [29]. Black powder performance is also highly dependent upon the strength with which it is confined [30]. When it is spread on the ground and lit in an open, unconfined environment it burns with negligible pressure generation; however, when it is confined the charge casing allows the gradual generation of internal pressure until the point of casing failure [32]. While it is categorized as a low explosive, the data presented in this study show that it is capable of generating a sharp-rising pressure wave with certain confinement conditions.

### TNT relative equivalency (RE)

TNT is conventionally used as a yardstick to compare the strengths of different explosives, both low and high, which are typically described by stating their TNT relative equivalency (RE) [33]. RE is a fractional number that describes the strength of an explosive relative to the strength of TNT. Most explosives have RE values in the range 0.8–1.2 [33, 34].

### Blast injuries

Conventional explosions can injure through one of three generic mechanisms, one of which is primary injury from the blast pressure wave itself (see Ref [35] for information on other injury types). Primary injuries from blast predominantly affect the gas-filled organs, most often causing pulmonary trauma such as hemorrhaging, but can also present as traumatic brain injuries [36, 37]. These injury types would not be evident from skeletal or highly decomposed remains.

## Methods

### Scale model construction

**Fig 2:**
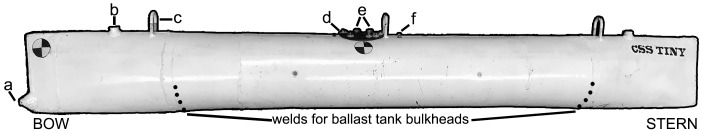
Photograph of the scale *Hunley* model, nicknamed the *CSS Tiny*. [a] threaded attachment for spar [b] access port (2 total, one each at bow and stern) to fill and empty the ballast tanks, can be sealed with threaded insert [c] Rings (3 on model) for carrying the vessel and attaching lines [d] Gasket-sealed panel for interior access [e] Data ports (2 on model) for gauges [f] Bulkhead fittings (4 on model) for gauge wires.

A 1/6 length-scaled model of the *HL Hunley* was constructed out of 16-gauge mild steel, which is materially similar to the wrought iron of the submarine’s hull in many properties including those that dictate structural response to blast exposure (S1 Table) [14, 38, 39]. Key physical design properties of the *Hunley* were incorporated in the scale model construction, including ballast tanks that could be filled with water and ballast weighting by lining the keel with lead. Information about the methods used to obtain the measurements of the *Hunley* that formed the basis of the scale model can be found in Ref [15]. The finished model is shown in Fig 2.

The *Hunley* model, nicknamed the *Tiny*, was exposed to underwater blasts via three primary experimental methods: shock tube exposures, black powder charges attached to the bow with a size-scaled, angled spar, and black powder charges directed at the side of the hull. For each exposure type, an omnidirectional incident pressure gauge was suspended in the center of the interior of the hull. An identical pressure gauge was also suspended in the water external to the boat, at the centerline along the length of the boat and at a distance of 6 cm horizontal standoff from the side of the hull. The gauges used were oil-filled tourmaline gauges validated for measurement of underwater and air blasts (Naval Surface Warfare Center Carderock Division, Bethesda, MD), amplified with a PCB Piezotronics 402A amplifier and powered with PCB Piezotronics model 482A10 and model 482 power supplies (PCB Piezotronics, Inc., Depew, NY). The gauge cables were foam-covered and insulated along the length of the cable between the boat and the pier. Data acquisition was performed at 1 MHz rate with 500 kHz antialiasing filters using a Hi-Techniques MeDAQ Win600e (Hi-Techniques Inc., Madison, WI).

The experiment was scaled so that the degree of pressure transmission into the scaled model hull would be similar to the degree of pressure transmission into the full-sized *Hunley* [17, 19, 21, 40–42]. Some changes, such as the change in density from salt to fresh water, were experimentally unavoidable, but the effects are expected to be small (differences in density and bulk modulus increase the speed of sound in salt water 2–4% over that in freshwater).

### Shock tube blasts

The shock tube exposures were performed in the Duke University Reclamation Pond. The shock tube was composed of a driver section only, made of size 3 high-pressure stainless steel pipe flanges, fitted with a variable number of Mylar membranes (S2 Fig). The shock tube driver was braced from behind with water-saturated wooden rails and pressurized with helium until the Mylar membranes ruptured, creating a shock wave. A live charge creates spherically expanding shock waves, so because the *Hunley’s* spar held the torpedo at a downward angle the *Hunley* would have been directly exposed from all sides along the entire length of its hull. Since shock tubes create highly directional shock waves rather than spherical shock waves like those produced by live charges, the shock tube was used to characterize which sections of the hull were responsible for transmitting the effects of blast. The characterization was performed by directing the shock tube at the bow of the vessel, at the side of the vessel, at the vessel’s keel, and at oblique angles relative to the axis of the hull. Once it was determined that the bow of the vessel transmitted negligible blast effects into the main cabin, and the perpendicular component of the blast was responsible for blast transmission into the cabin, the transmission tests were performed by directing the shock tube perpendicularly at the side of the hull. The external pressures were estimated to be the perpendicular component of a blast with the correct direction of propagation, and so were divided by sin(11°) to calculate the overall peak pressure values of the estimated blast [43].

### Live charge blasts

The test site for the live charges was a freshwater pond with a bottom depth slightly greater than the scaled value (9 m/6 = 1.3 m) of the bottom depth at the location of the *Hunley* attack on the *Housatonic* [44]. This depth would ensure that reflections of the blast waveform off the bottom would be equal to or less than those experienced by the *Hunley*, and so would either approximate the amount of bottom reflection or err on the low side since bottom reflections augment the strength of an underwater blast exposure [17].

All necessary permits and legal permissions were obtained prior to each round of testing. Charges were packed with 4Fg black powder (Goex Powder, Inc., Minden, LA) with casings constructed out of schedule 80 PVC pipe with threaded end caps. The historical drawing of the *Hunley* torpedo indicates that it was filled with grade FF cannon powder (S1 Fig). However, samples of powder from recently uncovered Union cannonballs from the Civil War indicate that the historic FF grain size more accurately matches the modern 4F grain size standard for musket powder [29]. The charges were triggered using NPb squibs (Martinez Specialties, Groton, NY). Isolated squibs were set off to evaluate their detonation signatures at the distances of interest, but were determined not to have a noticeable impact on the pressure waveforms.

Several preliminary underwater tests were conducted with variations in charge size, casing construction, and range to the point of measurement to assess black powder’s performance with respect to the scaling of time constant for the non-dimensional groups. Charge size was varied, with sizes of 283 g, 455 g, 490 g, and 1 kg, and range between the charge and the point of measurement was varied between 80 cm and 1.8 m. The range values were selected to validate the scaling principles in the regions most relevant to the *Hunley*. Initial testing showed that orientation and position of the charge relative to the gauges had a measurable effect on the pressure waveforms. Therefore, test data were only used for the analysis of scaling if they had the same charge orientation and depth as the tests with the scale boat model, and gauge locations that would fall along the length of the submarine hull. Specifically, the tests evaluating the effects of confinement strength were eliminated from the scaling data set because the measurements were taken at the same depth as the charges.

The initial time constant of decay (θ) was measured for all blasts. Theta was scaled using Hopkinson scaling by division by the cube root of charge weight (W^1/3^) (Eq (2)). This value was then plotted as a function of the scaled distance (W^1/3^/Range) at which the waveform was measured. A power law equation was fitted to the data in the scaling data set using least-squares regression. This method is the standard procedure for describing the time-scaling behavior of explosives and is referred to as the principle of shock wave similitude [17, 21].

The *Tiny* model was blasted with black powder charges of three sizes: 283 g and 455 g charges, corresponding to 1/6 and 1/5 size scale of the 61.4 kg (135 lb) *Hunley* torpedo, and 1 kg charges, which were the maximum size as requested by the ATF. While 283 g is the properly mass-scaled value for black powder, the larger charge sizes were constructed to evaluate the degree of propagation of higher pressures through the hull wall. Experimental limitations on scaling burn rate of the powder and methods of charge confinement meant that the PVC 283 g charges would severely underestimate the strength of the exposure compared to the original copper-cased torpedo; larger charges were therefore also tested to evaluate how the transmission properties changed with increases in external pressure. All charges except one were attached via a size-scaled spar to position them in the same manner as the *Hunley’s* torpedo. One 283 g charge was positioned beside the boat to further increase external pressure of exposure. The external pressure from this charge was divided by the sine of the angle between the direction of blast propagation and horizontal at the centerline of the keel (11°) to calculate the total external peak pressure from a spar-mounted charge that would have the same amount of propagation through the hull. This angular correction for direction of transmission is often used in structural shock testing for charges in different geometric orientations from their targets [43].

### Shock tube blasting of metal plate

Propagation of a sharp-rising shock wave through the full-sized *Hunley* structure was investigated using a mild steel plate with greater thickness (1.6 cm, 5/8”) than the original *Hunley* hull (1.0 cm, 3/8”). The purpose of this test was to ensure that the shock wave maintained the sharp rise time critical to cause injuries even when propagating through a material at least the thickness of the full-sized submarine hull. The steel plate was a square 61 cm (24”) on each side and was exposed to airblast using a helium-driven shock tube. The shock tube was 30.5 cm (12”) in diameter and was aimed at the center of the plate. A standoff distance of 4 cm was set between the plate and the end of the tube to allow lateral venting of the shock and provide reduced impulse on the plate relative to peak incident pressure. Incident pressure was measured at the end of the shock tube using 200 psia Endevco pressure gauges (Model 8530B-200, Meggitt Sensing Systems, Irvine, CA) that were flush with the internal wall of the tube body. Two additional Endevco pressure gauges were rigidly fixed behind the center of the steel plate, with 10 cm between the back of the plate and the center of the gauge faces. Both gauges were oriented to measure incident pressure.

### Data analysis

Data were low-pass filtered at 40 kHz. Tests of blast injury within an enclosed environment have shown that the magnitude of peak pressure of a complex, non-ideal waveform with a locally rapid rise time is associated with injury risk, regardless of when during the waveform that peak occurs [45, 46]. Therefore, the internal boat peak pressure was determined to be the peak pressure of the waveform that was achieved via a rapid (<2 ms) rise time, even if the peak pressure did not occur at the beginning of the waveform. Ratio of transmitted pressure was calculated using peak pressures from the external and internal pressure waveforms.

Previous groups examining blast transmission into a structure have analyzed the ratio of impulse transmitted to the structure relative to the incident external impulse (I_t_/I_i_) and developed empirical curves to describe the observed pattern of behavior in air [22, 24, 26, 47, 48]. The equations for these curves are dependent on the magnitude of external incident shock pressure normalized by ambient pressure (P_s_/P_0_). In other words, an increase in external pressure also results in an increase in the *percentage* of that pressure that is transmitted into the structure. However, these equations were developed for shock waves in air using the ideal gas equation, and the increase in transmission is partially a result of the increase in reflection coefficient (C_R_) at the surface with increasing pressure ratios. The decreased compressibility of water means that low-pressure shocks reflect with behavior approximating the simple doubling of peak pressure of acoustic waves [17]. For example, an underwater shock with an incident overpressure of 100 MPa has a reflection coefficient of only 2.088 [17, 49]. The *Hunley* explosion was estimated to have peak pressures far below 100 MPa (confirmed by experimental results, see Results and Discussion), so a reflection coefficient at the acoustic limit of C_R_ = 2 was determined to be a reasonable approximation. The original equation derived by Kambouchev et al. to describe the ratio of impulse transmitted into a plate (I_P_) relative to impulse of the incident shock (I_i_) is shown below as Eq (5).

$$\frac{I_{P}}{I_{i}}=\gamma_{R}{\left(\frac{C_{R}f_{R}}{\gamma_{R}}\right)}^{\beta_{s}/\left(1+\beta_{s}\right)}\beta_{s}{}^{\beta_{s}/\left(1-\beta_{s}\right)}$$ (5)

β_s_ = pi group value (Eq 4)

f_R_ = nondimensional factor

γ_R_ = relative transmitted impulse at the heavy plate limit

Where γ_R_ and f_R_ are dependent upon the overpressure ratio P_s_/P_0_ [24, 47, 48]. However, if the shock reflects with C_R_ = 2 then γ_R,_ the limit for behavior with a heavy plate, equals the reflection coefficient C_R_ (Ref [24], Eq 46). The factor f_R_ is derived using the ideal gas equation to examine transmission of impulse, and therefore the analytical equation cannot be correctly applied to the behavior of pressure transmission in water. A full derivation of f_R_ in water is beyond the scope of this publication; however, this factor shows relatively little fluctuation even for air, and approaches a value of 1 at the acoustic limit (maximum 1.26, approaches 1.13 as P_s_/P_0_→∞) [24]. Therefore, this parameter was fit to the experimental data by minimizing sum of least squares error using the empirically derived form of Eq (6) (8.1.0.604, TheMathWorks, Inc., Natick, MA) [24].

$$\frac{Transmitted}{Incident}=C_{R}{\left(b\right)}^{\beta_{s}/\left(1+\beta_{s}\right)}\beta_{s}{}^{\beta_{s}/\left(1-\beta_{s}\right)}$$ (6)

b = fit parameter

Initially multiple curve fits were performed by grouping the data according to peak overpressure ratio P_s_/P_0_ (two groups with values ranging 1–2 and 3–5, median 1.2 and 3.2 respectively). However, the fit lines for each group fell within the confidence intervals for the other group. It was therefore determined that a single unified curve should be fit to all the data, as the possible variation from f_R_ was not sufficient to cause statistically significant changes in the resultant transmission curves for this dataset. Sensitivity analysis was also performed to determine the sensitivity of the qualitative conclusions on variations in C_R_.

The actual percentage of the peak pressure that was transmitted for the *Hunley* explosion was obtained from the curve fit at the calculated β_s_ value for the *Hunley* explosion. The transmission percentages from the 95% confidence intervals were used as upper and lower bounds. However, the tests discussed herein exposed the scale model to charge amplitudes, and therefore P_s_/P_0_ ratios, smaller than those estimated to have occurred from the full-sized *Hunley*. Therefore, the percent transmitted during the real blast should be greater, but without a full characterization over a wider range of P_s_/P_0_ ratios it is difficult to quantify how much greater.

The injury risk assessment from Panzer et al, 2012 for pulmonary fatality is the most appropriate because it has the largest body of experimental backing and specifically evaluates long-duration airblasts, defined as overpressures longer than 10–30 ms [50]. They and others concluded that for this blast exposure type, injury risk is primarily determined by peak overpressure and has little duration dependence [50–52]. Per scaling principles, the durations of overpressure in the scale model should be multiplied by a factor of 6 following the initial peak to represent the durations of the full-scale *Hunley*. The durations for the complex waveform types seen inside the vessel hull are difficult to conclusively measure, but previous work examining blast in enclosed spaces has determined that these complex waveforms are equivalent to a “quasi-static pressure increase” that injures comparably to a long-duration blast with the same peak overpressure value [53]. The waveforms seen experimentally showed durations of at least 10 ms for all tests; with scaling, this is equivalent to a duration of at least 60 ms for the *Hunley* crew. Longer durations would have a higher associated risk. While this is a conservative assumption, the relative insensitivity of risk level to exact duration value for long-duration blasts also means that the results change little if a longer-duration assumption was made. The guidelines from Rafaels et al, 2012 for risk of fatality from primary blast traumatic brain injury (TBI) were also used to assess the risk of fatality to the crew [36]. However, it should be noted that these guidelines are based on a dataset with mass-scaled durations of 15 ms or less; therefore, a duration of only 15 ms was used to calculate risk to avoid overestimation of risk for long-duration blasts.

**Fig 3:**
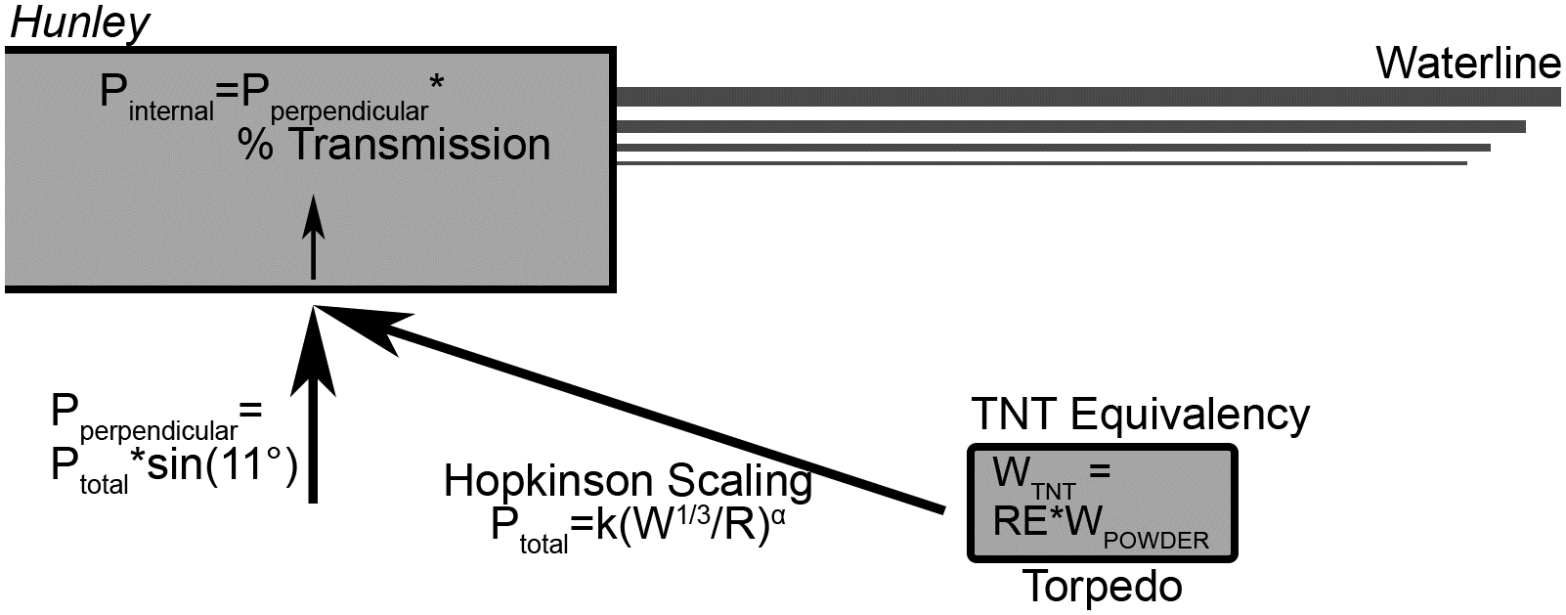
Diagram of the *Hunley* and charge, illustrating the steps of the analyses using relative equivalency (not to scale). Analysis was performed both starting with known black powder equivalencies and calculating risk to the crew, and also by starting with the minimum internal pressure required to cause fatality and calculating necessary TNT equivalency.

The risks of injury and fatality were estimated using two related but independent methods. A diagram of the methods is shown as Fig 3 for clarity, in addition to being described in the text.

Previous studies have shown that though black powder deflagrates rather than detonates, when it is used in confined casings, high explosive scaling laws (TNT scaling) accurately predict charge output [54]. While the relative equivalency (RE) for black powder can vary based on exact experimental setup and level of charge confinement, values in the literature for confined black powder were found to be within the range of 0.24–0.46 (median value 0.43) [34, 55, 56]. The range of equivalency values were applied to the known *Hunley* charge weight of 61.4 kg to calculate the range of estimated equivalent charge weights of TNT. Hopkinson scaling laws were then used to calculate the peak pressure resulting from such a blast at the central point of the vessel’s keel (range R = 10.8 m). The peak pressure was multiplied by the sine of the angle between the direction of blast transmission and horizontal (11°) to determine the component perpendicular to the surface of the keel [43]. The experimentally determined transmission percentages were used to calculate the peak pressure that would have been transmitted to the interior crew cabin. This peak pressure was then used, with a duration estimate of 60 ms, to calculate the risks of injury and fatality to the crew [36, 50].

The second method of analysis performed the same steps but in reverse to calculate the minimum required RE necessary for the crew to avoid injury or fatality. Risk was calculated as a function of peak overpressure for a 60 ms duration blast using the curves from Panzer et al. [50]. The experimentally determined percent transmission levels were then used to calculate the peak pressures required of the external blast exposure for sufficient transmission to cause those levels of risk. This external blast exposure was assumed to be the component perpendicular to the hull, and was therefore divided by an angular correction factor (sin(11°)) to calculate the total pressure. Hopkinson scaling was used with the required total external peak pressure levels to calculate the weight of TNT that would create those pressures in water at the distance of the center of the *Hunley*’s keel (Eq (1)). Dividing the required TNT charge weight by the known weight of the *Hunley*’s black powder charge resulted in the minimum TNT relative equivalency that would be necessary for the black powder to produce at least that level of airblast exposure inside the *Hunley*. Calculated REs that were atypically low compared to the measured values for black powder could then be considered to support the theory that the *Hunley* torpedo created a blast wave at least strong enough to cause injury and fatality to the crew inside.

## Results

**Fig 4:**
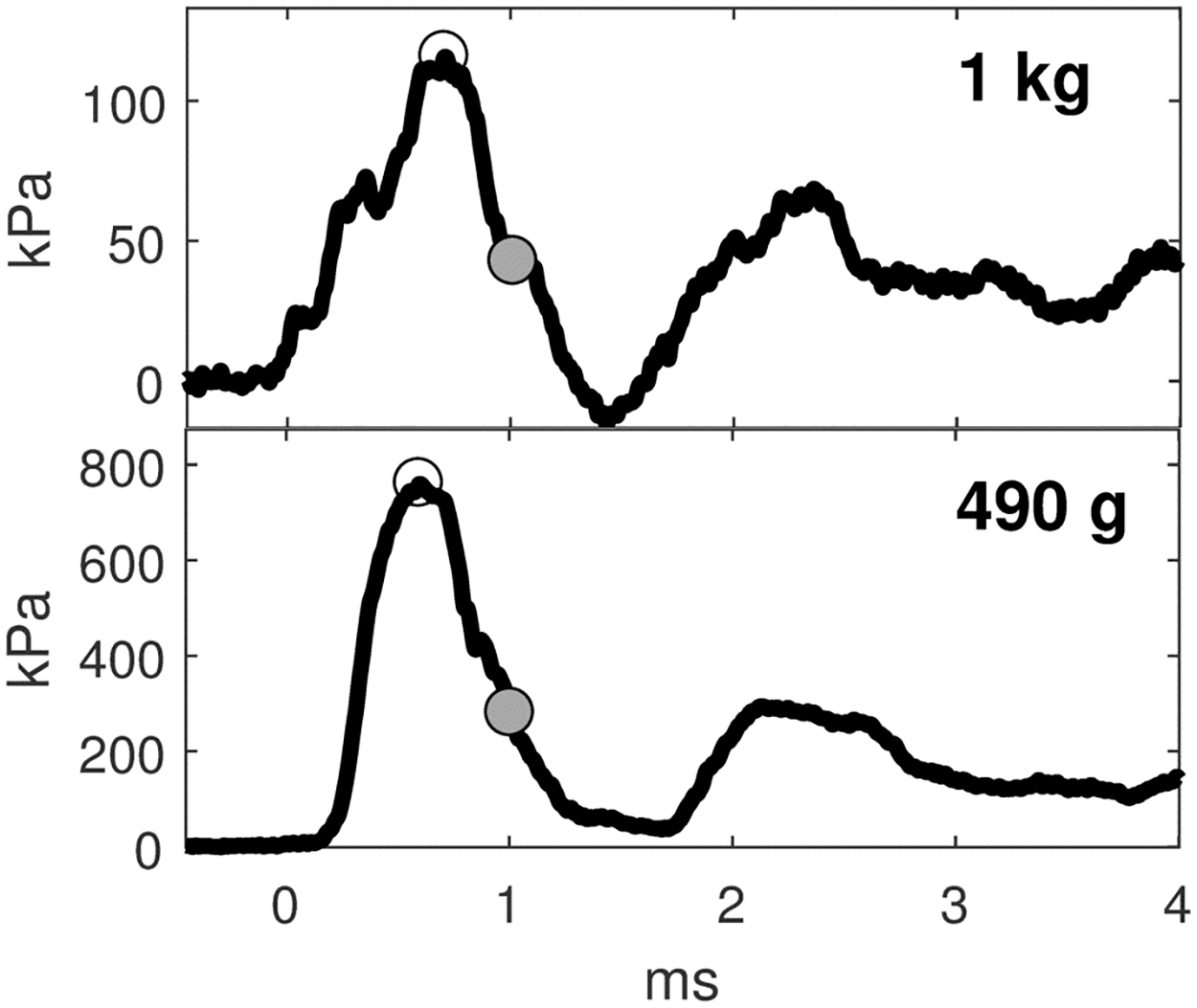
Representative curves of initial blast wave from black powder charges of two sizes. The open circle indicates the peak of the wave, and the grey circle indicates one time constant of decay. The 1 kg charge was at a range of 1.8 m with a time constant of 265 μsec. The 490 g charge was at a range of 0.8 m with a time constant of 408 μsec.

Preliminary tests showed that the charge output was dependent on charge orientation, degree of confinement, and strength of the charge casing (S3 Fig). Representative curves of the initial blast waves from two different charge sizes are shown as Fig 4.

**Fig 5:**
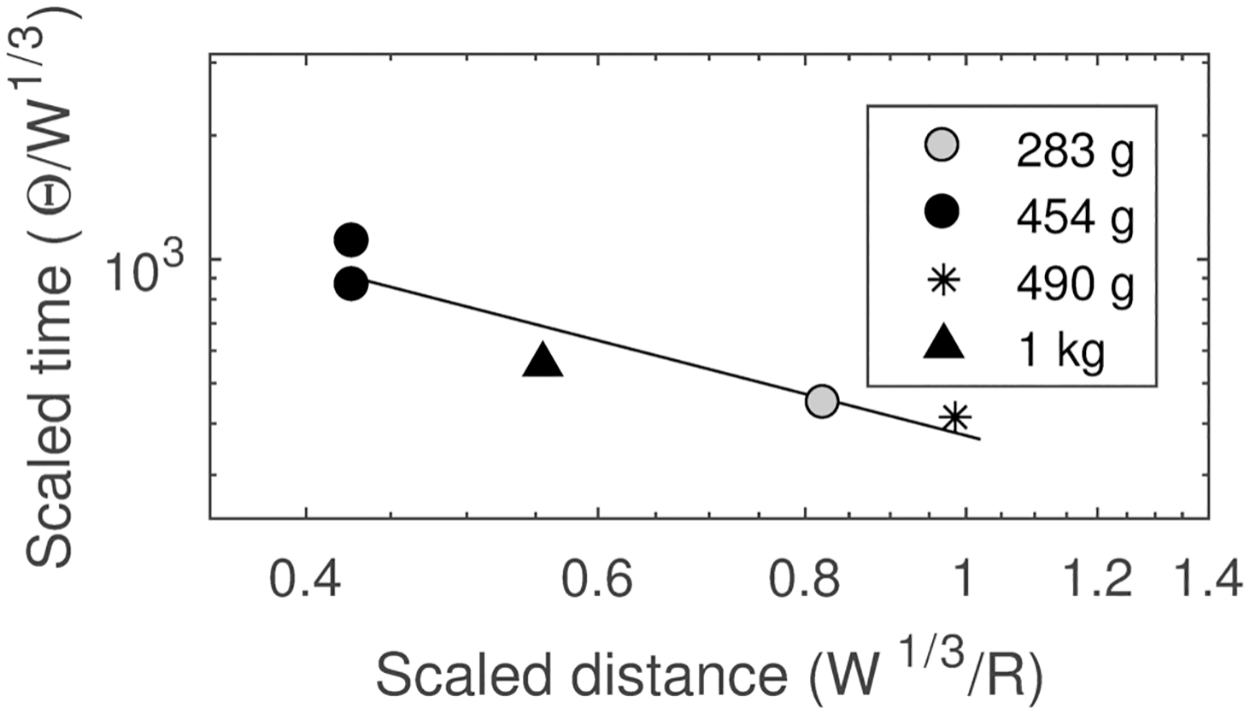
Scaled time constant as a function of scaled distance. The data for black powder show the power law trend consistent with other known explosive types.

The scaled time constants (θ/W^1/3^) are shown plotted against scaled distance (W^1/3^/R) in Fig 5.

The resulting curvefit equation is shown below as Eq (7), which was used to calculate time constants for the *Hunley* explosion. The equation showed an R^2^ = 0.87 fit with the data.

θW13=373.7(W13R)−1.039 (7)

θ = time constant of initial decay (μsec)

W = charge weight (kg)

R = distance from charge (m)

The values of the pi groups for both the scale model and the full-sized *Hunley* explosion are calculated in S2 Table. The scaled model shows a decrease from the full-size *Hunley* explosion of 6.5% in the Taylor pi group value (Eq 3) and 2.8% in the Kambouchev et al pi group value (Eq 4) [23, 24].

**Fig 6:**
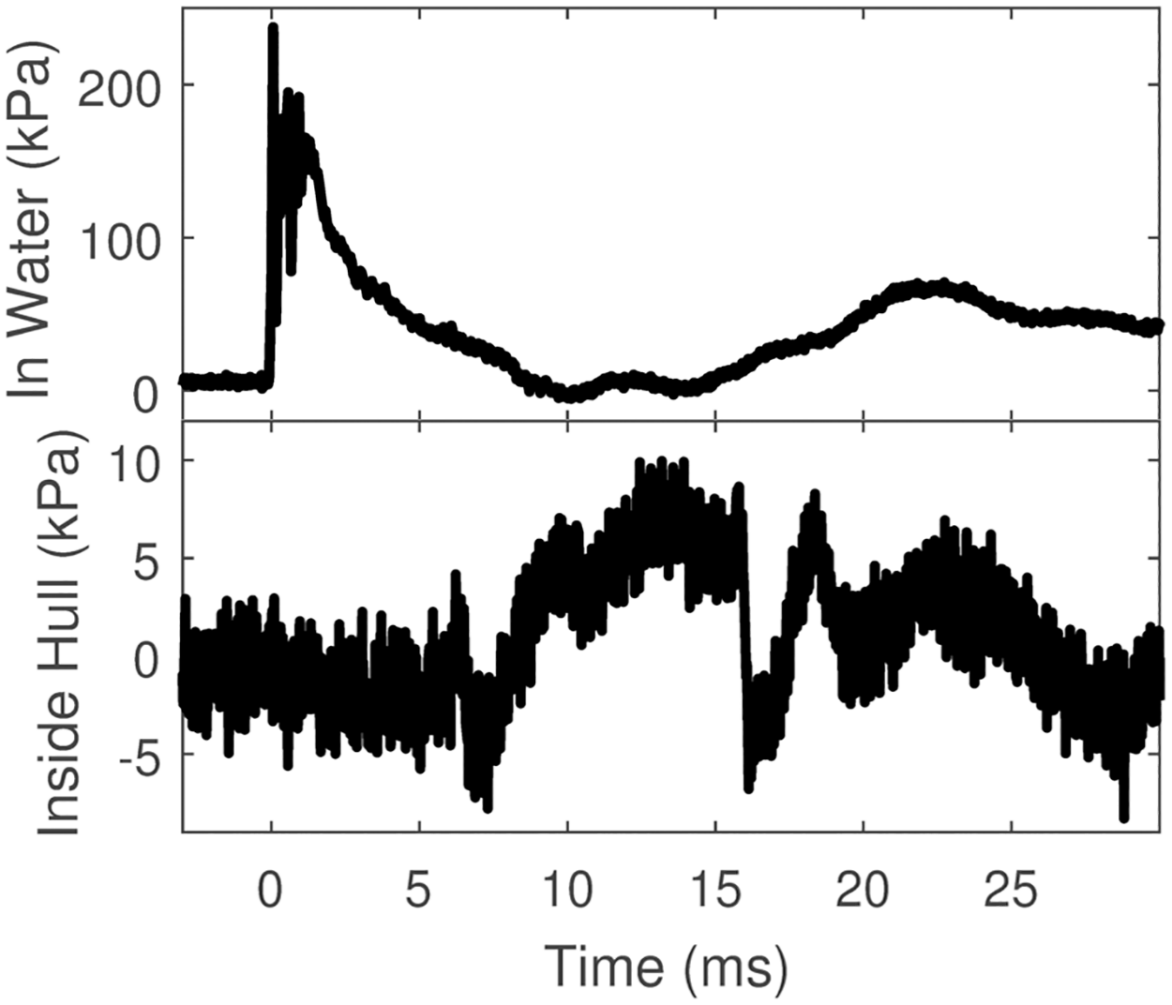
Representative waveform showing transmission into the scale model. This test had a shock tube orientation perpendicular to the side of the scale model. [a] Waveform in water [b] Waveform inside the boat hull.

Representative waveforms showing transmission from the shock tube through the side of the boat are shown as Fig 6.

**Fig 7:**
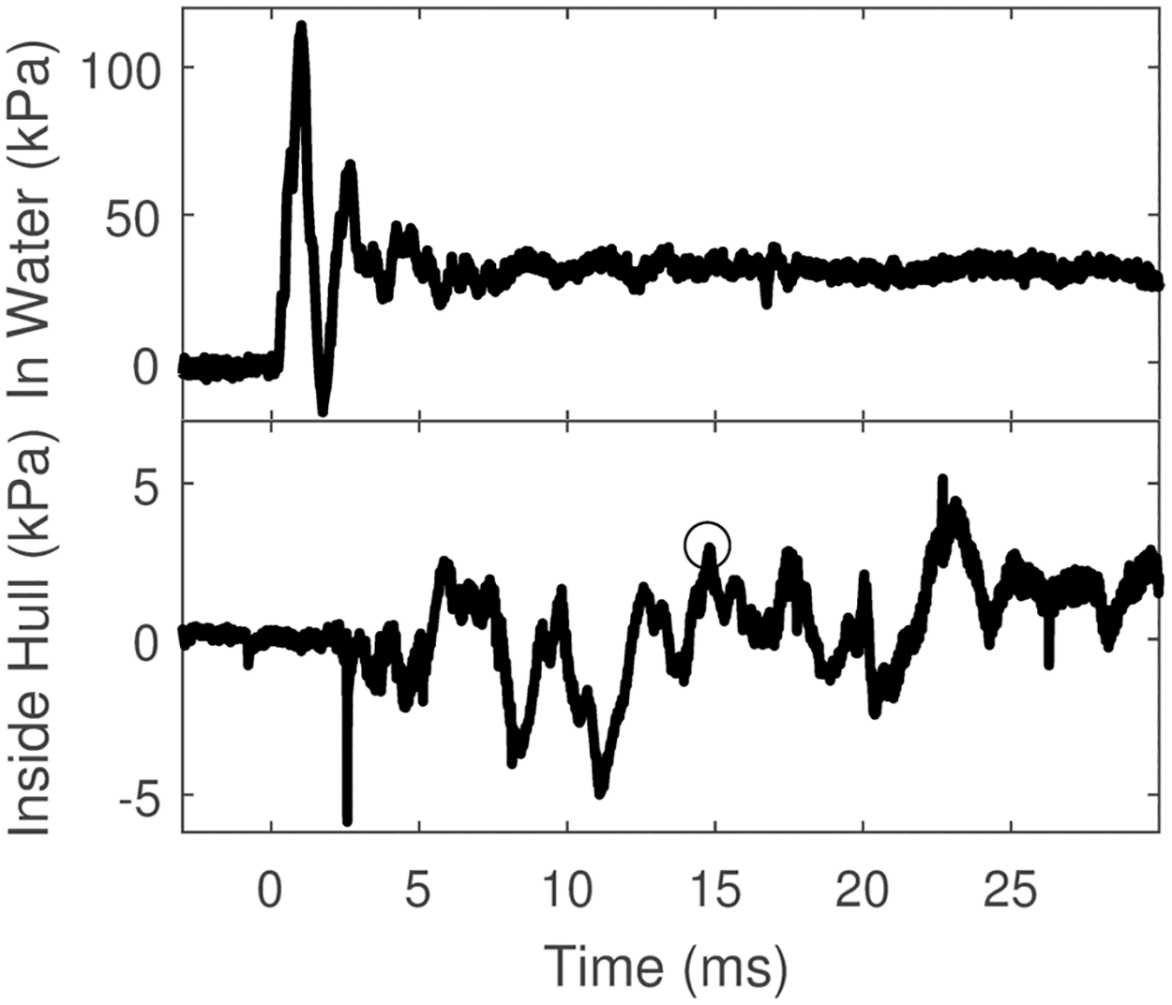
Blast transmission into the model hull of 1 kg charge on a spar. The circle in the lower panel indicates the selected point of peak pressure (defined as peak pressure achieved with <2 ms local rise time).

Fig 7 shows a waveform for a test of blast transmission into the hull of the scaled boat from a 1 kg black powder charge at the spar position.

The peak pressure of the external waveform was 109 kPa (15.8 psi), with a perpendicular component of 20.7 kPa. The peak pressure of the internal waveform was 2.94 kPa (0.43 psi), a 14.2% transmission.

**Fig 8:**
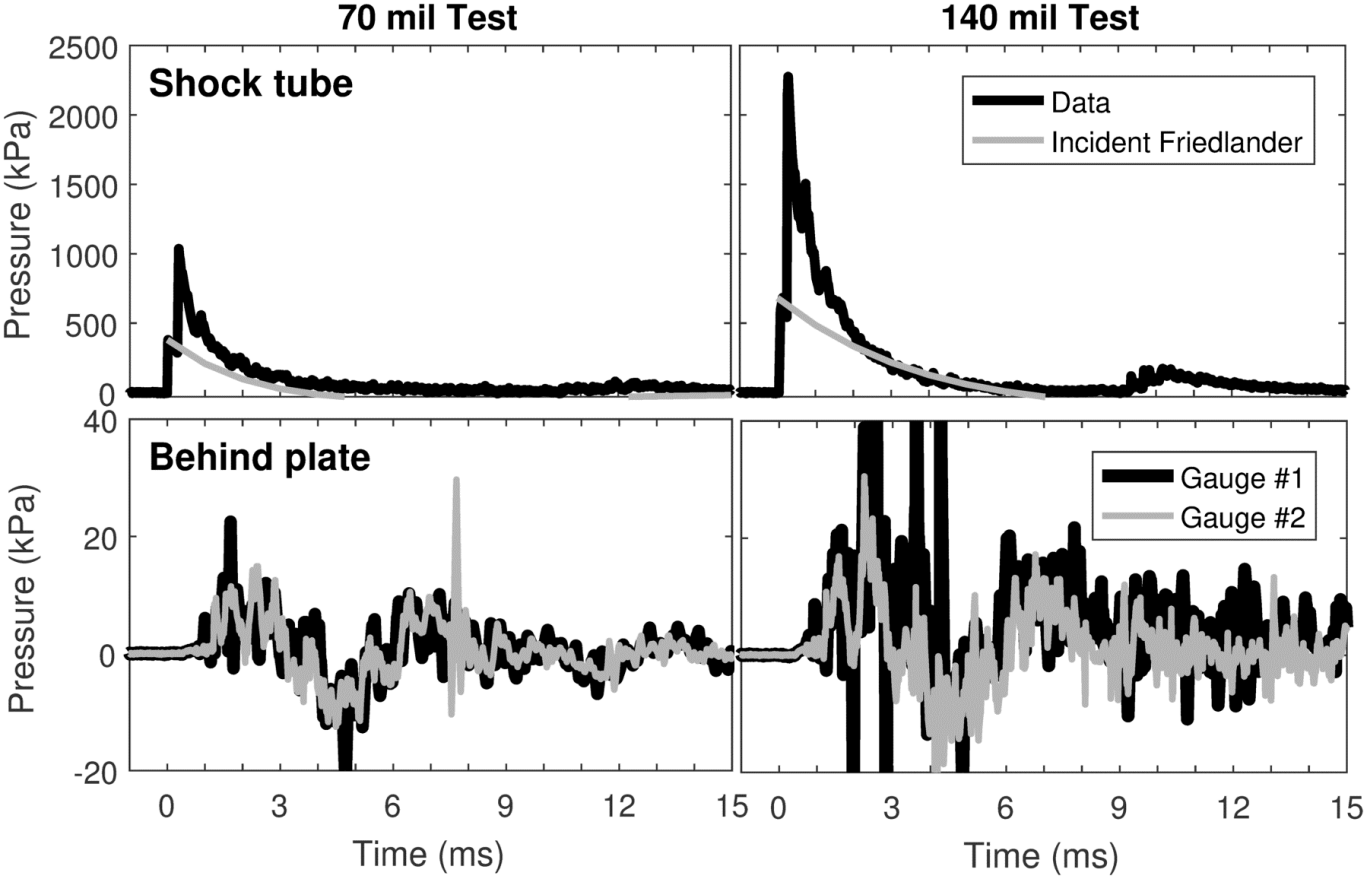
Waveforms transmitted through the 1.6 cm (5/8”) thick mild steel plate. Shock tube waveforms have been overlaid with the ‘external’ incident pressure Friedlander curves produced by these shock tubes in this configuration without the reflection back into the tube from the steel plate. Gauge 1 in the 140 mil tests was dislodged during the test and shows some spike-shaped anomalies between 2–5 ms.

The blasts of the metal plate showed transmission of a shock wave with a sharp rise time at both pressure levels of exposure. The waveforms are shown in Fig 8. Both plate tests showed a definite pressure increase with a sharp (<1 ms) rise time.

**Fig 9:**
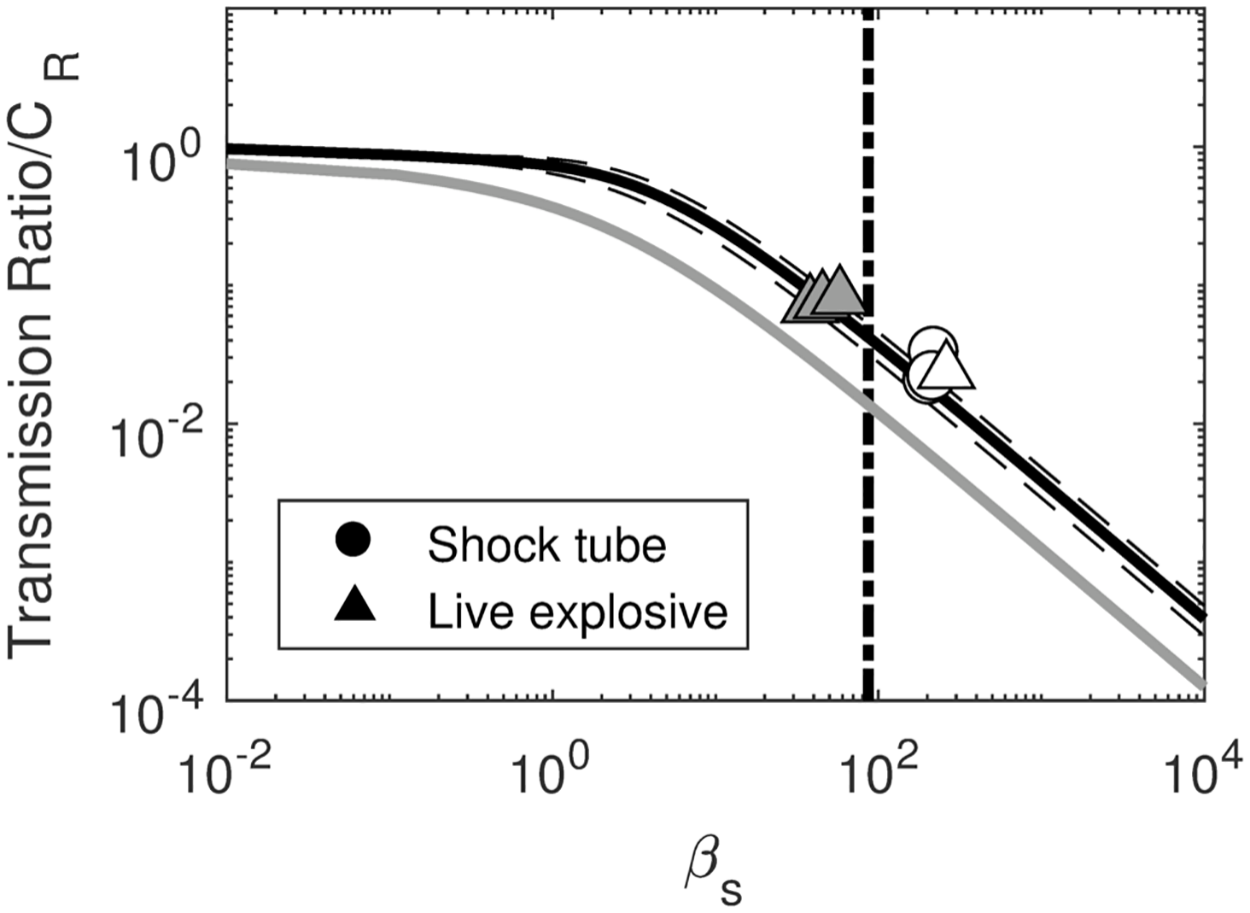
Ratio of peak pressure propagating through the wall [24, 47]. Dashed lines show 95% confidence intervals. The vertical line indicates the β_s_ value calculated for the *Hunley* explosion. The calculated transmitted impulse is shown in grey for reference; axes selected for consistency with previous works [22, 24, 26, 47, 48].

The live charge blasts of the ship model had curve fit values of b = 2.82 (Eq 6). The curve is shown with the data in Fig 9, with the transmitted impulse curve also shown in grey for reference.

The transmission rate for the β_s_ of the *Hunley* explosion was 8.4%. However, the transmission value is still lower than anticipated to have occurred during the actual 1864 blast because the P_s_/P_0_ ratio would have been higher than was achieved experimentally; therefore both the level of exposure and the rate of transmission would be higher. The 95% confidence intervals were used to find the upper and lower bounds for transmission (6.3% and 10.5%). Variation of the C_R_ values within a factor of 4 (maximum physically possible) showed a change in the calculated percent transmissions by less than 0.1%; this variation was within the bounds of the calculations used to draw qualitative conclusions.

**Fig 10:**
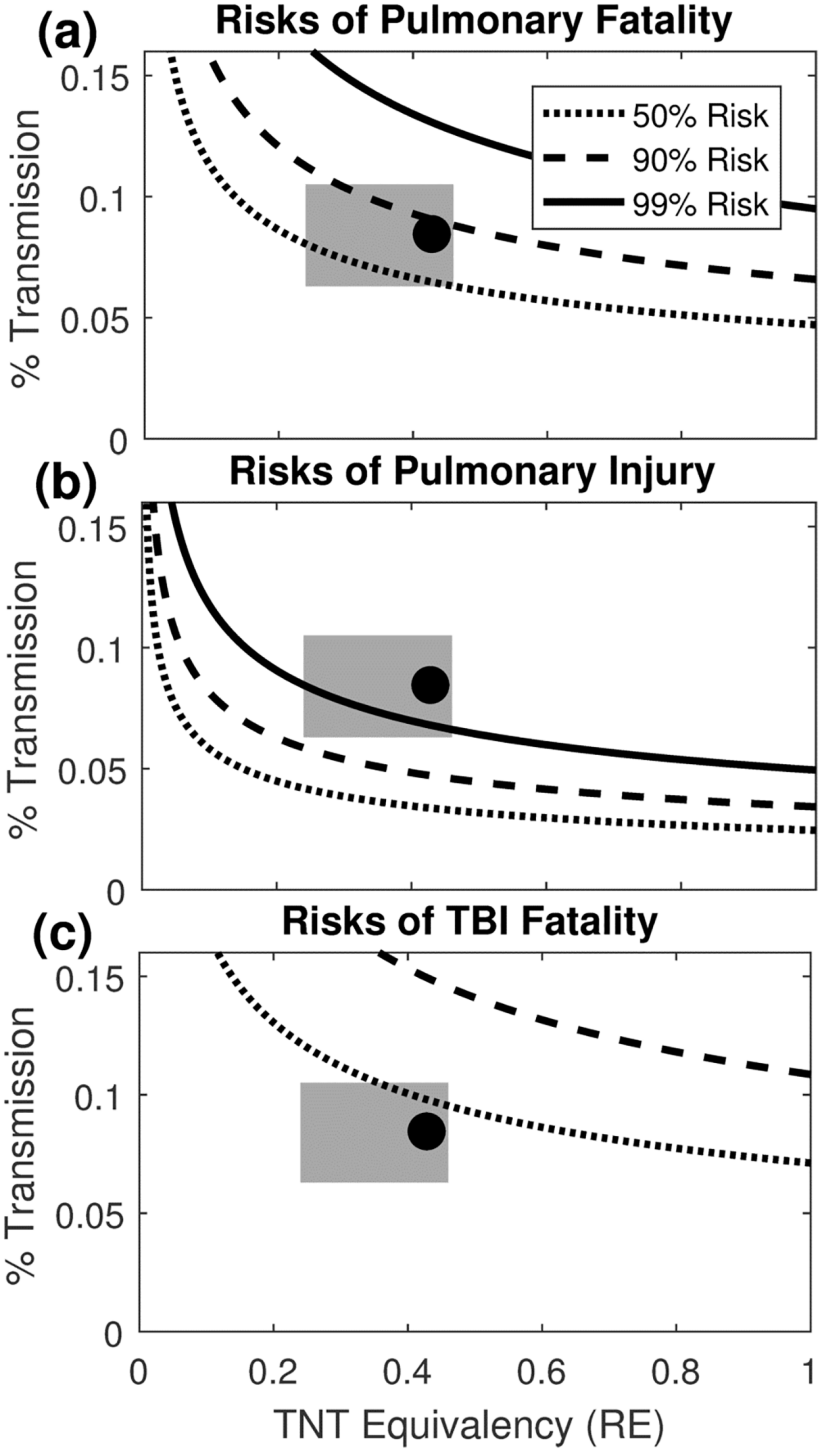
Risks of injury and fatality for a range of TNT relative equivalencies and transmission levels. Grey rectangles indicate the range of physically reasonable values (RE [0.24, 0.46]; transmission [6.3%, 10.5%]) (a) Risk of pulmonary fatality [50] (b) Risk of pulmonary injury [50] (c) Risk of fatality from traumatic brain injury [36]. The exposure to the *Hunley* crew (closed circle) was calculated using a 8.4% transmission level and RE = 0.43, the median value found in the literature (see Methods). This exposure is a low estimate because 8.4% was the transmission occurring at the low experimental overpressure ratio of 3.4, and rate of transmission would continue to increase up to the P_s_/P_0_ = 68 expected of the full-sized explosion (value of 68 justified in the Discussion).

The results of the calculations relating TNT equivalency to injury and fatality (described in Fig 3) are shown graphically in Fig 10.

The lower bound of experimentally measured black powder equivalencies (0.24) results in a calculated 9.8 MPa exposure outside the hull, 157 kPa inside the hull, and 57% risk of pulmonary fatality from airblast at the 8.4% transmission level. The median RE for black powder (0.43) resulted in 12.2 MPa outside the hull, 195 kPa inside, and 85% risk of pulmonary fatality at 8.4% transmission. Performing the analysis from the reverse direction concludes that with the calculated 8.4% transmission level, an RE of 0.21 or lower is required for less than a 50% chance of pulmonary fatality to the crew; this value is lower than most measured RE values for explosives [33]. An RE of 0.09 or lower is required for less than a 90% risk of pulmonary injury at the same transmission level (Fig 9b). The risks of fatality from traumatic brain injury are 31% even assuming the shortened duration of 15 ms, as shown in Fig 10c. The *Hunley* exposure, with an RE of 0.43 and calculated transmission of 8.4%, results in a 85% chance of pulmonary fatality despite using the pressure transmission rates for the experiments with lower peak external pressures than the actual event.

## Discussion

The black powder charges used were calculated to be underpowered compared to the estimated output of the *Hunley’s* torpedo. Preliminary tests confirmed that the strength of the blast wave produced by black powder is sensitive to the strength of the external casing (S3 Fig). This result is consistent with the known performance characteristics of deflagrating low explosives [57]. Unconfined or minimally confined black powder deflagrates slowly enough that it generally does not produce a sharp-rising wave on casing failure. The presence of sharp-rising waves in the underwater experimental data indicates that the peak pressures achieved were the result of the sudden failure of the casings and the subsequent detonation-like release of the gaseous products [58]. Therefore, it is unlikely that the burn rate of the black powder played a critical role in determining the pressure output, and that the pressure output was instead dependent upon the strength of the charge casing.

Historical data of black powder manufactured during the late 1800s showed that a 61.4 kg black powder charge, when confined, reached a peak internal pressure of 167,000 kPa (24,200 psi) [59]. The transmission of at most 336 kPa into the water during this study indicates it is highly unlikely that the modern experimental charges reached this better-confined value of 167,000 kPa. Conservation of the *Hunley’s* spar revealed that the copper torpedo shell was forced backwards in ribbons over the end of the spar, indicating that the copper torpedo casing had sufficiently confined the black powder charge to cause a uniform explosion (S4 Fig). In contrast, the casings for the experiments herein were found to have failed by plastic deformation of the end cap threads, indicating that the end caps failed prior to buildup of sufficient pressure necessary to uniformly rupture the casing.

Therefore, it is likely that the charges that were used for this study output peak pressure values lower than the pressures produced by the *Hunley’*s charge, potentially by up to a factor of 64 based on the 2.6 MPa rated failure pressure of the construction materials and the historical data [59, 60]. US Navy testing of a full-sized black powder charge designed to measure the output of the *Hunley*’s torpedo showed peak pressure values of approximately 7,600 kPa (1,100 psi) at a measurement location comparable to the keel of the *Hunley* [*unpublished data*]. This pressure is a factor of 43 increase over the maximum 176 kPa peak pressure measured along the model hull for the tests herein, confirming that the charges used for the blast tests with the scaled model were less strongly confined in comparison to the charge of the full-sized *Hunley*. A peak overpressure of 7,600 kPa at the depth of the *Hunley*’s keel yields a P_s_/P_0_ ratio of 68, far higher than the ratio of 3.2 from the higher-pressure group of the tests of these studies. This increase in pressure would increase the percentage of transmission; however, it is unclear how much.

The results from the shock tube and live charge tests show that the transmission behavior of peak pressure is consistent with the transmission behavior of impulse. Impulse transmission has been validated to maintain the described behavior up to peak exposures of at least 1.35 MPa using experimental methods [22] and at least 101 MPa using computational methods [26]. For a constant wall thickness, any increase in momentum transfer must present as an increase in wall velocity, and compressible fluid mechanics dictates that increasing wall velocity results in exponentially increasing peak overpressure for the shock wave induced behind the plate [25]. Therefore, while the rate of transmission of peak pressure has not been expressly validated at the higher pressures expected of the *Hunley* blast, peak pressure is directly linked to impulse through wall velocity, and impulse transmission has been validated in these pressure regions. It should also be noted that while a strain gauge (Micro Measurements Model C2A-03062LR-350) placed on the inside of the hull consistently showed deformation coincident with the arrival time of the blast wave, the strain measurements were subject to experimental complications and therefore the proposed mechanism of deformation-induced transmission remains speculative for this case.

The ratios of transmitted peak pressures show that the charge of the *Hunley* could transmit sufficient blast levels inside the vessel to cause a high risk of injury and fatality to the crew. It is important to note that risk levels are not sensitive to whether the exposure occurs from the side or the front of the torso, so if the airblast propagated inward at an angle relative to the length of the submarine’s cylindrical axis rather than perpendicular to it, the risks of fatality would be the same for the crew.

A common misconception is that people exposed to blast are always physically thrown by the blast (e.g. in movies or television). However, blasts too weak to move or translate a human body noticeable distances are still often intense enough to cause lethal pulmonary trauma [45]. For the *Hunley*, since the hull was exposed from all radial directions simultaneously and accompanied by motion of the ship itself, there may be no clear direction of motion even if the pressure wave did translate the crewmen. Lethal pulmonary blast injuries are therefore consistent with the lack of skeletal trauma and the positions of the crew at their battle stations.

Respiratory distress is one of the hallmarks of pulmonary blast injury; even if any crewmen had survived the initial blast they would have likely still been above the injury threshold and would have experienced symptoms such as shortness of breath, hemoptysis, tachypnea, and hypoxia [16, 45, 61]. Therefore, even if some crewmen had survived the initial blast they would have likely been crippled in terms of respiration and physically unable to power the handcrank to move the submarine. If anyone had survived, they may have tried to release the keel ballast weights, set the bilge pumps to pump water, or tried to get out the hatches, but none of these actions were taken.

Eyewitness reports stated that they saw a blue light on the water after the attack, the *Hunley’*s pre-arranged symbol for victory [2, 62]. However, eyewitness reports are notoriously unreliable especially in the heat of battle, as evidenced by the *Housatonic* crew’s inability to agree on either the level of the tide or direction of the current at the time of the attack [63]. *Based on the analysis above*, *the crew was instantly killed by primary blast trauma*.

## Conclusion

This work has several limitations. The largest limitation is weak charge confinement owing to the construction of the scaled charges. This inability to achieve pressure levels comparable to the levels expected from the full-sized charge means that the experiments herein likely underestimate the actual blast transmission. However, the pressure values transmitted inside the boat demonstrated that even at these low blast levels, sufficient transmission would have occurred to cause fatal pulmonary or brain trauma to the crew.

A second major limitation is that the equations to calculate the transmission of impulse to flat surfaces assume, in the final steps of their derivation, that the surrounding medium is air. For a surface with water on one side and air on the other, the final forms of these equations must be re-derived if it is desired to calculate the exact impulse values imparted to the metal hull. Then the impulse and resultant plate motion must be combined with the laws of compressible fluid mechanics to calculate the exact pressures that would have been experienced by the crew. However, the purpose of this study was not to determine analytical equations to describe the exact pressure level for all such blasts, but rather to draw qualitative conclusions about the fate of the crew. For the purposes of this study, this limitation has therefore been addressed by fitting the form of the equations to the data and performing a sensitivity analysis. The quantitative data strongly suggest that the whole crew was instantly killed by airblast trauma, and the sensitivity analysis suggests that this conclusion is insensitive to moderate changes. All test data are available online via PLOS One in S1 File.

The Pyrrhic attack of *H*.*L*. *Hunley* was responsible for the deaths of 21 Confederate crewmen and 5 Union sailors. An earlier Confederate vessel that successfully used a spar torpedo, the *David*, was made of wood and floated much higher in the water. Additionally, earlier designs of the *Hunley* had the submarine towing the torpedo on the surface of the water far behind its stern, and were successfully used in trial runs in the harbor [2]. It was the combination of all the simultaneous design changes: conversion from wood to wrought iron, sinking the vessel deeper in the water, lowering the torpedo, and attaching the charge much closer at the end of a spar that ultimately led to the demise of the crew. The *H*.*L*. *Hunley* presents the first documented case of primary blast-induced fatality to personnel within a structure.

## Supporting information

S1 Fig Historical drawing of the *Hunley’s* torpedo. This drawing, found in historical documentation, shows a drawing of the torpedo “used for blowing up the Housatonic.” The drawing is done to scale, and shows the details of the angled attachment to the spar, the pressure-sensitive trigger mechanism. It also specifies that the torpedo was packed with 135 lbs of black powder.
(JPG)

S2 Fig Shock tube driver. Pressurization of the driver section leads to rupture of the Mylar membranes and creation of a shock wave. This picture was taken after a test, and the ruptured membranes can be seen in the center of the opening. The fill port is on the reverse side. The ropes were used to raise and lower the driver into the water.
(PNG)

S3 Fig Outputs of 100 g charges with varying levels of confinement. (PNG)

S4 Fig Scientific illustration of the *Hunley*’s spar, showing the torpedo remnants. The 61.2 kg (135 lb) black powder charge had a thick copper shell, and when the *Hunley*’s spar was conserved, portions of the torpedo shell were still present. The method of attachment is consistent with the archival drawings of the *Hunley*’s torpedo (S1 Fig). The copper shell shows damage from the explosion both in the remnants that have peeled backwards over the spar, and also because the entire shell was pushed backwards and the attachment bolt cut through the shell.
(PNG)

S1 Table Material properties of wrought iron and mild steel. Mild steel is similar to wrought iron in the material properties most critical to proper replication of the effects of blast transmission.
(DOCX)

S2 Table Values for pi groups that determine blast transmission. Water temperatures were measured at 13°C for the scaled experiment, and estimated as 10°C for the *Hunley* explosion. 10°C is the mean temperature outside Charleston Harbor in February [S2 Table Ref 1]. Material values from S1 Table. Speed of the blast wave was not directly measured and has been estimated as the speed of sound in water, shown to be a sufficient approximation at the pressure levels relevant to these exposures [S2 Table Ref 2].
(DOCX)

S1 File All test data. This file contains all the test data used for the conclusions presented in this publication.
(ZIP)
