# Supplementary figures and images for: Air blast injuries killed the crew of the submarine H.L. Hunley
Source: PLoS One. 2017 Aug 23;12(8):e0182244. doi: 10.1371/journal.pone.0182244 (PMC5568114; doi:10.1371/journal.pone.0182244)

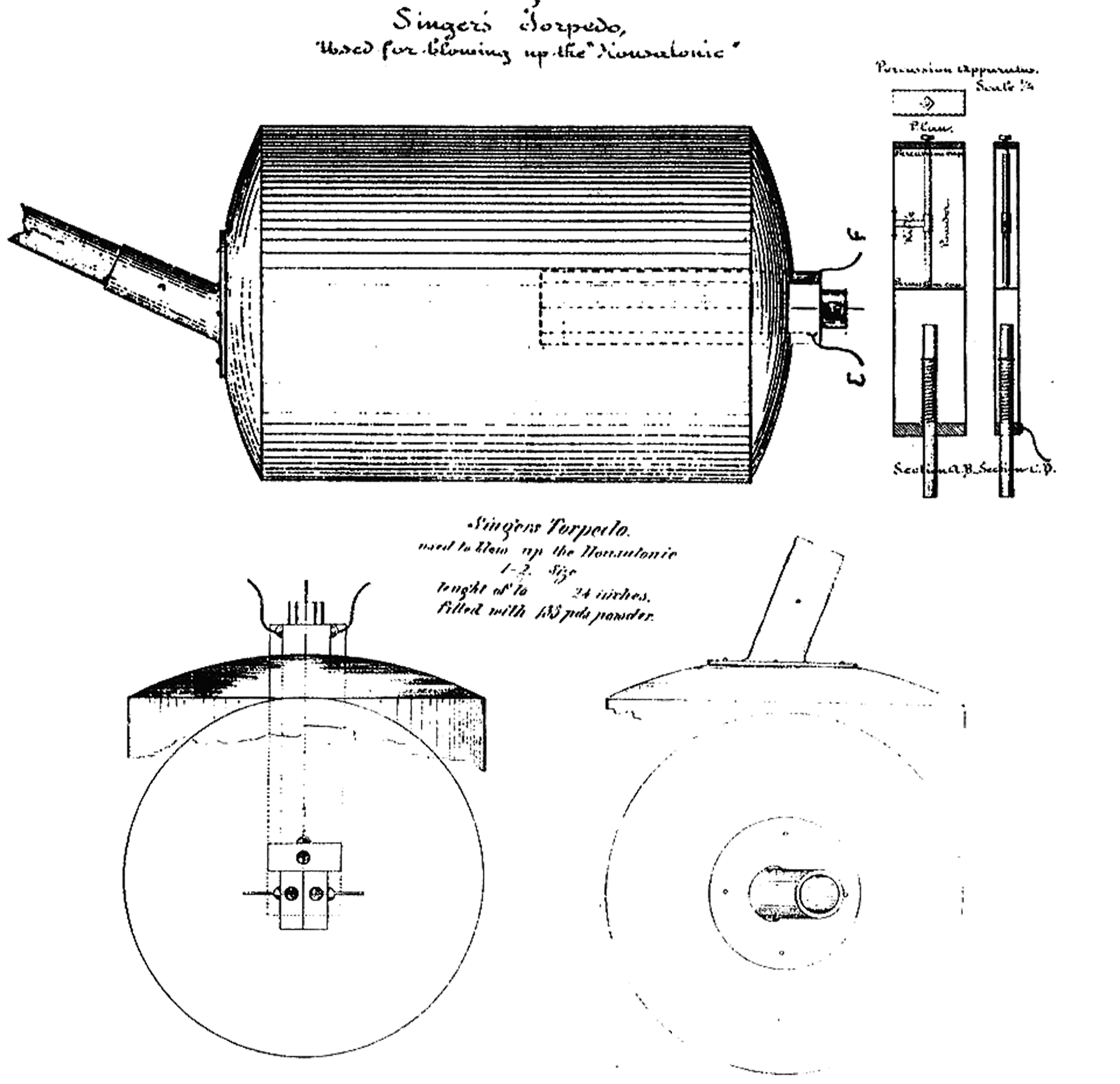

Supplement: S1 Fig — This drawing, found in historical documentation, shows a drawing of the torpedo “used for blowing up the Housatonic.” The drawing is done to scale, and shows the details of the angled attachment to the spar, the pressure-sensitive trigger mechanism. It also specifies that the torpedo was packed with 135 lbs of black powder. (JPG) [file pone.0182244.s001.jpg]

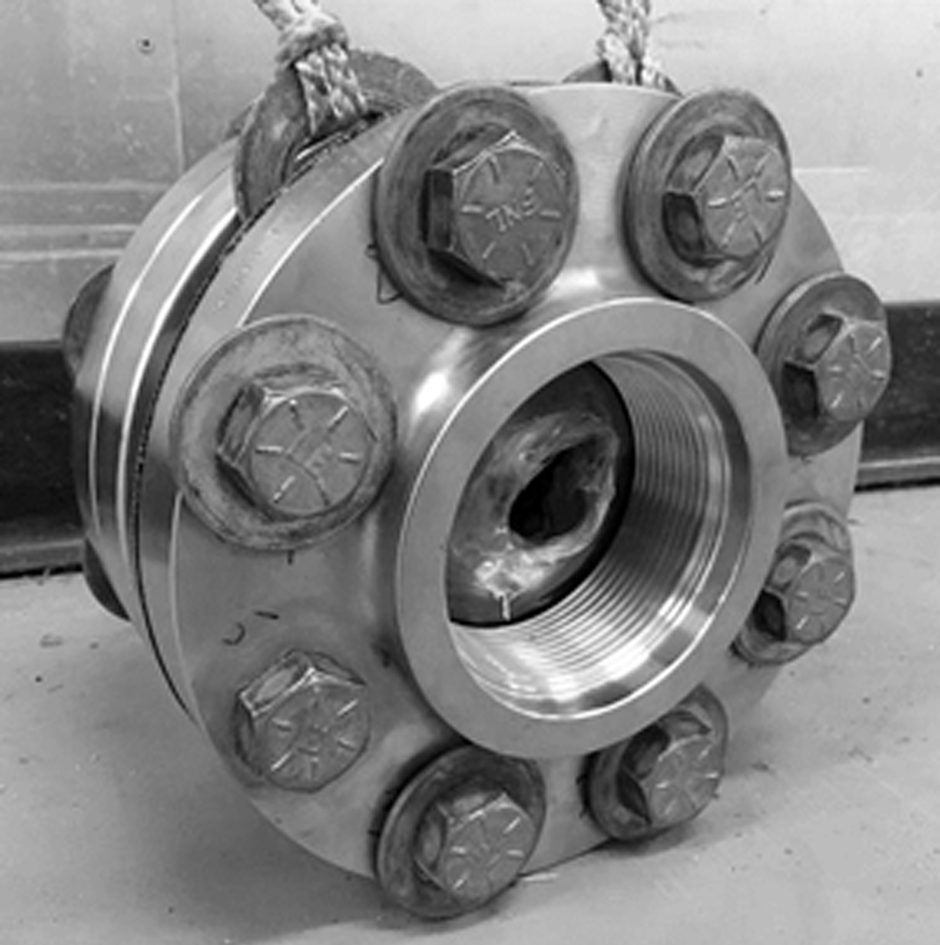

Supplement: S2 Fig — Pressurization of the driver section leads to rupture of the Mylar membranes and creation of a shock wave. This picture was taken after a test, and the ruptured membranes can be seen in the center of the opening. The fill port is on the reverse side. The ropes were used to raise and lower the driver into the water. (PNG) [file pone.0182244.s002.png]

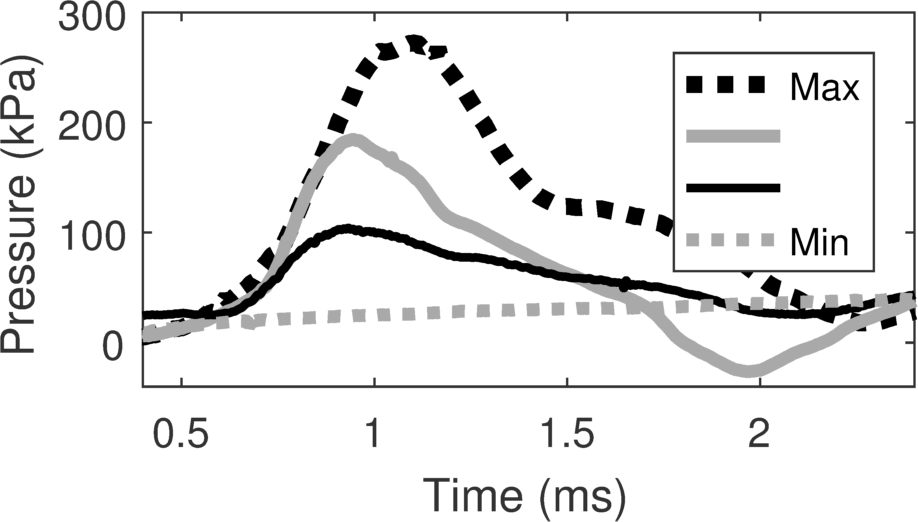

Supplement: S3 Fig — (PNG) [file pone.0182244.s003.png]

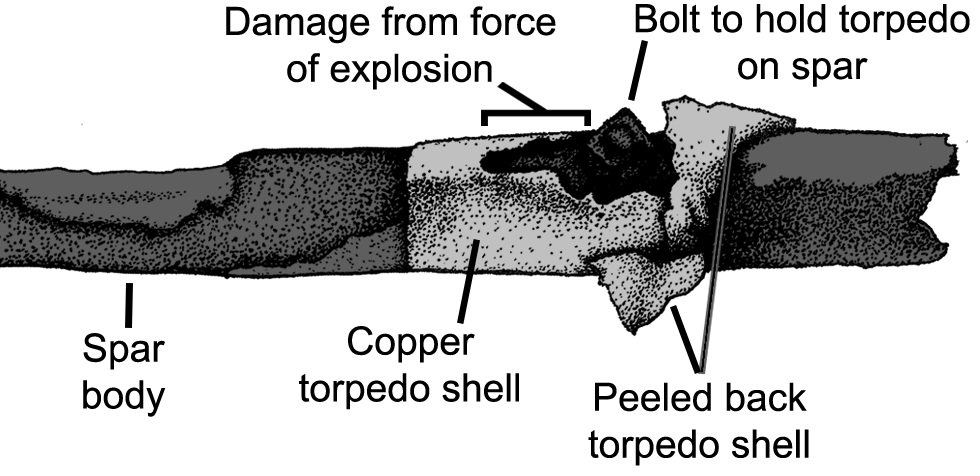

Supplement: S4 Fig — The 61.2 kg (135 lb) black powder charge had a thick copper shell, and when the Hunley’s spar was conserved, portions of the torpedo shell were still present. The method of attachment is consistent with the archival drawings of the Hunley’s torpedo (S1 Fig). The copper shell shows damage from the explosion both in the remnants that have peeled backwards over the spar, and also because the entire shell was pushed backwards and the attachment bolt cut through the shell. (PNG) [file pone.0182244.s004.png]

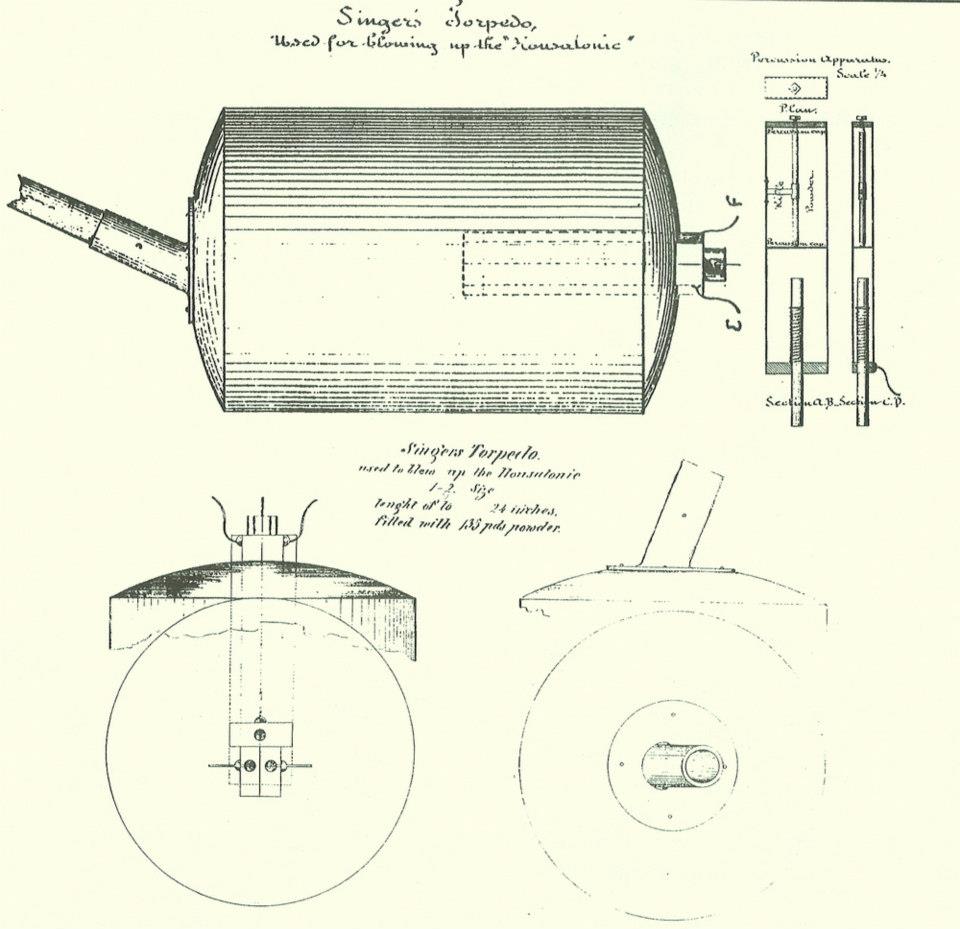

Supplement: S1 File — This file contains all the test data used for the conclusions presented in this publication. (ZIP) [file pone.0182244.s007.zip › Lance2017_HunleyData/Torpedo_original_diagram.jpg]
