# Supplementary material for: Air blast injuries killed the crew of the submarine H.L. Hunley
Source: PLoS One. 2017 Aug 23;12(8):e0182244. doi: 10.1371/journal.pone.0182244 (PMC5568114; doi:10.1371/journal.pone.0182244)
Supplement: S1 Table — Mild steel is similar to wrought iron in the material properties most critical to proper replication of the effects of blast transmission. (DOCX) [file pone.0182244.s005.docx]

**Table S1. Material properties of wrought iron and mild steel.** Mild steel is similar to wrought iron in the material properties most critical to proper replication of the effects of blast transmission.

|  | Wrought Iron | Mild Steel | % Change |
| --- | --- | --- | --- |
| Density  (ρ) (kg/m^3^) | 7,677 (1) | 7,833 (2) | +2.0 % |
| Speed of sound  (c) (m/s) | 5,056 (3) | 5,050 (3) | -0.1 % |
| Impedance  (z) (kg/(m^2^*s)) | 3.88 x 10^7^ | 3.96 x 10^7^ | +2.1 % |
| Modulus of elasticity (E) (GPa) | 196 (1) | 210 (4) | +7.1 % |
| Bulk modulus  (κ) (GPa) | 160 (5) | 159 (6) | -0.6 % |

**REFERENCES**

1. Barker GF. Physics: advanced course. 3rd ed. New York, NY: Henry Holt and Company; 1892.

2. Mamalis AG. Finite element simulation of chip formation in orthogonal metal cutting. Journal of Materials Processing Technology. 2001;110(1):19-27.

3. Avadhanulu MN, Kshirsagar PG. A textbook of engineering physics. New Delhi, India: S. Chand and Company LTD; 1992.

4. Sarkar BK. Strength of Materials: Tata McGraw-Hill Education; 2003.

5. Parasnis DS. Seismic methods. Principles of Applied Geophysics. Berlin, Germany: Springer Science and Business Media; 1986. p. 250-317.

6. Fragomeni S, Venkatesan S. Incorporating sustainable practice in mechanics and structures of materials. Boca Raton, FL: CRC Press; 2010.
