# Supplementary material for: Air blast injuries killed the crew of the submarine H.L. Hunley
Source: PLoS One. 2017 Aug 23;12(8):e0182244. doi: 10.1371/journal.pone.0182244 (PMC5568114; doi:10.1371/journal.pone.0182244)
Supplement: S2 Table — Water temperatures were measured at 13°C for the scaled experiment, and estimated as 10°C for the Hunley explosion. 10°C is the mean temperature outside Charleston Harbor in February [S2 Table Ref 1]. Material values from S1 Table. Speed of the blast wave was not directly measured and has been estimated as the speed of sound in water, shown to be a sufficient approximation at the pressure levels relevant to these exposures [S2 Table Ref 2]. (DOCX) [file pone.0182244.s006.docx]

Table S2. Values for pi groups that determine blast transmission. Water temperatures were measured at 13° C for the scaled experiment, and estimated as 10° C for the *Hunley* explosion. 10° C is the mean temperature outside Charleston Harbor in February (1). Material values from Table S1. Speed of the blast wave was not directly measured and has been estimated as the speed of sound in water, shown to be a sufficient approximation at the pressure levels relevant to these exposures (2).

| **Variable** | **Scaled Experiment** | **Full-Sized *Hunley* Explosion** |
| --- | --- | --- |
| ρ, ρ_s_ = density of medium in front of the structure (kg/m^3)^ | 1000 | 1026  (3) |
| c = speed of sound in medium in front of the structure (m/s) | 1498  (4) | 1531  (4) |
| m = areal density of the structure (kg/m^2^) | 12.4  (scales with L) | 73.1 |
| n = inverse time constant of decay of the blast wave (1/seconds) | 1.85 e +3  (From Eq (7))  (scales with 1/L) | 3.07 e +2  (From Eq (7)) |
| ϕ = angle of the structure surface, if conical | 90° | 90° |
| U_s_ = propagation speed of the incident blast wave | 1498 (approximated as speed of sound) | 1531 (approximated as speed of sound) |
| t_i_ = time constant of the incident blast wave (seconds) | 5.42 e -4  (From Eq (7))  (scales with L) | 3.25 e -3  (From Eq (7)) |
| ρ_p_ = mass density of the structure (kg/m^3^) | 7677 | 7833 |
| h_p_ = structural thickness (m) | 1.59 e -3 (1/16”) | 9.53 e -3 (3/8”) |
| Taylor pi group value | 82.6 | 88.4 |
| Kambouchev pi group value | 84.3 | 86.7 |

**TABLE S2 REFERENCES**

1. NOAA. Water temperature table of the southern Atlantic coast 2016 [Available from: www.ngdc.noaa.gov.

2. Cole RH. Underwater explosion. New York, N.Y.: Dover Publications, Inc.; 1948.

3. Karleskint G, Turner R, Small J. Introduction to Marine Biology. Boston, MA: Cengage Learning; 2012.

4. CRC. CRC Handbook of Chemistry and Physics. 37 ed. Ohio: Chemical Rubber Co.; 1968.
